# Polio vaccination campaigns in conflicts: succeeding while other humanitarian efforts fail?

**DOI:** 10.3389/fpubh.2025.1600755

**Published:** 2025-07-03

**Authors:** Majdi M. Sabahelzain, Hazem Agha, Nadav Davidovitch, Oliver Razum

**Affiliations:** ^1^Sydney School of Public Health, The University of Sydney, Sydney, NSW, Australia; ^2^Sydney Infectious Diseases Institute, Faculty of Medicine and Health, The University of Sydney, Sydney, NSW, Australia; ^3^Al-Quds Public Health Society, Jerusalem, Palestine; ^4^School of Public Health, Ben-Gurion University of the Negev, Be'er Sheva, Israel; ^5^School of Public Health, Bielefeld University, Bielefeld, Germany

**Keywords:** polio, water sanitation and hygiene (WASH), war, Israel, Palestine

## Abstract

In conflict settings, public health interventions such as vaccination campaigns and improvements in water, sanitation, and hygiene (WASH) could benefit all parties involved. However, while polio vaccination campaigns frequently succeed in securing humanitarian pauses, WASH initiatives attempting to improve safe water supply and sewage disposal rarely achieve the same outcome. Using the Israel-Gaza conflict as an example, we analyze the factors contributing to the success of polio vaccination campaigns compared to WASH initiatives. We identify four key elements that facilitate the implementation of polio campaigns in conflict zones: (i) the ubiquitous decline in vaccine coverage and the subsequent detection of polio cases; (ii) international institutional support, including the role of the Global Polio Eradication Initiative (GPEI); (iii) the shortness of the required humanitarian pauses, the vertical nature, and the straightforward impact assessment of vaccination campaigns; and (iv) their “neutral” character due to an intentionally restrictive focus on children as the primary beneficiaries. Although polio vaccination campaigns do not typically lead to lasting peace and WASH initiatives often fail to secure even temporary humanitarian pauses, public health efforts should seize every opportunity to foster cooperation between warring parties. Such initiatives can help build trust, laying the groundwork for future peace negotiations and post-conflict reconstruction.

## Introduction: public health measures benefit all

Many public health measures benefit not only those populations which are directly targeted, but also other populations in their vicinity. For example, vaccination against childhood diseases will not only protect children who are immunized; given a sufficiently high immunization coverage, herd immunity sets in, protecting also the small proportion of children who did not receive vaccination for various reasons, including medical and religious exemptions. The protective effect achieved extends even further: a high immunization coverage in one area makes it less likely that an infection will be imported into a neighboring area. An analogous argument can be made for providing safe water, hygiene, and sanitation (WASH) measures: they will reduce the risk of disease outbreaks not only locally, but also in neighboring areas—if there are fewer contagious agents in one place, they are less likely to spread to another place.

Armed conflicts and wars disrupt the provision of public health measures such as routine immunization programmes. Moreover, military action frequently destroys—as “collateral damage” or intentionally—public health infrastructure. This includes health centers, vaccine storage facilities, but also water pipes and sewage processing plants. In wars that are being fought between neighboring, densely populated countries, all adversaries should have an interest in avoiding the spread of communicable disease, as it may also affect their respective population and combatants. Taking this idea further, public health measures that prevent the spread of disease could firstly provide reason for humanitarian pauses in fighting, or even ceasefires, to allow vaccination campaigns; and secondly help ensure that conditions for civilians in war-affected areas fulfill or surpass minimum humanitarian standards, as for water and sanitation. The Israel-Hamas war in Gaza demonstrates that this rationale only partly holds. Declining vaccination coverage and spread of vaccine-preventable diseases have been among other deteriorating public health indicators as repercussions of armed conflict. Nevertheless, polio vaccination campaigns are among the few interventions that have succeeded to convince parties in this and other armed conflicts to engage in an immediate and sufficiently extensive humanitarian pause to facilitate safe and unhindered delivery and distribution of vaccines.

Using the Israel-Gaza conflict as our main example, we analyze the factors contributing to the success of polio vaccination campaigns compared to WASH initiatives. We chose WASH because of the high risk of children to die from various diseases linked to unsafe water and poor sanitation; because of the attention it received already earlier in this conflict (1); and because UNICEF and other organizations explicitly speak of “WASH for peace” (2). We identify four key elements that facilitate the implementation of polio campaigns in conflict zones: (i) the ubiquitous decline in vaccine coverage and the subsequent detection of polio cases; (ii) international institutional support, including the role of the Global Polio Eradication Initiative (GPEI); (iii) the shortness of the required humanitarian pauses, the vertical nature, and the straightforward impact assessment of vaccination campaigns; and (iv) the “neutral” character due to an intentionally restrictive focus on children as the primary beneficiaries.

## The challenges and opportunities of implementing humanitarian pauses

To understand how humanitarian aid, including vaccination campaigns, is delivered in a conflict context, we must first distinguish different types: international war and intra-state or civil war, as well as acute and protracted conflict. In acute humanitarian crises like international war, WHO and UNICEF lead health interventions, including vaccination campaigns, coordinated by OCHA, the United Nations Office for the Coordination of Humanitarian Affairs (3). In areas affected by protracted conflicts and controlled by non-state armed groups (NSAGs), delivering health services, including vaccination, is complex. First, this is because of security challenges. Second, while NSAGs are the *de facto* sovereigns, UN agencies are mandated to respect the continued dominant role of the internationally recognized regimes in their *de jure* sovereignty (4).

To implement vaccination campaigns in protracted conflicts in NSAG-controlled areas, UN agencies cooperate with international non-governmental organizations (NGOs) that have access to and are trusted by local communities (5). For instance, the global Vaccine Alliance (Gavi) launched the Zero-Dose Immunization Program (ZIP) in 2022 to address vaccination inequities for vulnerable children in 11 conflict-ridden Sahel and Horn of Africa countries. Zero-dose means they are unvaccinated with any dose of diphtheria, tetanus and pertussis (DTP) containing vaccine. ZIP is run by two consortia, led by two international NGOs, World Vision and the International Rescue Committee, respectively (6). Recently, Gavi provided USD 23.4 million to support the immunization efforts of UNICEF and Save the Children during the ongoing conflict in Sudan, in which a large proportion of health facilities are non-operational. UNICEF focuses on accessible areas under the control of the government, whereas Save the Children focuses on reaching children in remote regions controlled by the Rapid Support Forces (RSF), a NSAG (7).

Polio vaccination campaigns have also been implemented successfully in active conflicts. For example, in 2024, the international community, including the United Nations (UN), repeatedly urged Hamas and the Israeli government to agree on a ceasefire in Gaza to protect war-affected civilians and deliver essential aid. The Israeli government, however, rejected these proposals until all hostages taken on October 7, 2023, were released. Nevertheless, the adversaries accepted two humanitarian pauses unrelated to the hostage situation. They were implemented to provide two rounds of polio vaccination after circulating variant poliovirus type 2 (cVDPV2) was detected in Gaza wastewater samples in July 2024, linked to a strain last seen in Egypt in 2023. By August 2024, a 10-month-old child was paralyzed by polio. Short humanitarian pauses allowed vaccination campaigns in September and November to reach more than 550,000 children, improving their protection against polio and supplying many of them with vitamin A (8); WHO recommends vitamin A supplementation in malnourished children and considers vaccination campaigns as safe and effective vehicles (9). It was rightly argued that vaccination campaigns benefit populations on all sides of a conflicts, and substantial hurdles were overcome to implement the campaigns (10, 11).

The logic pertaining to polio immunization should apply to water and sanitation measures in a similar way. In a geographically close area like Palestine, Israel, and Jordan, water, sanitation, and hygiene (WASH) issues do not remain local, and all sides would benefit from cooperation. Children in particular would benefit from high immunization coverage as well as from access to clean water in sufficient quantities. However, efforts to use improving WASH as a starting point for ceasefire negotiations in Gaza have stalled (1, 12).

## Why do polio campaigns succeed?

We first analyze in more detail what makes polio vaccination campaigns likely to work in conflict settings, before contrasting them to less successful efforts using WASH as a starting point for humanitarian pauses.

### Declining vaccination coverage and polio outbreaks in conflict settings

Armed conflicts and wars tend to have rapid and significant effects on vaccination coverage, even before severe infrastructural damage occurs. In 2023, 21 million children were identified as “zero-dose” or under-vaccinated globally, with 20% of them residing in conflict zones (13). Armed conflicts, and even preceding political instability, have been associated with dramatic declines in immunization coverage and repeatedly with the detection of polio cases, as in Sudan (14), but also in upper-middle income countries, as in Ukraine (15, 16). An analysis of vaccination coverage data from 16 conflict-affected countries found that almost 67% of global polio cases from 2010 to 2015 were reported from these countries (17). In Syria, the proportion of children vaccinated with DPT1 declined from pre-conflict levels of 89% in 2010 to 73% and 60% in 2012 and 2013 (18), following a full-scale civil war involving multiple factions, namely the Syrian government and NSAGs, including extremist groups like the Islamic State of Iraq and Syria (ISIS). Thirty-six cases of wild poliovirus type 1 (WPV1) have been reported in the country in 2013–14, caused by the importation of a strain of WPV1 circulating in Pakistan (19). Additionally, 74 cVDPV2 cases were reported in 2017 (20). In Ukraine, a cVDPV2 outbreak was detected in two young children in 2021, following the importation of poliovirus that had emerged in Pakistan and was previously detected in Tajikistan in 2021. An additional 19 close contacts tested positive without developing symptoms (21). Since Russia's full-scale invasion of Ukraine, vaccine coverage in Ukraine declined for example for the DTP-containing vaccine, from 91% in 2021 pre-invasion to 78% in 2022. Armed conflicts and wars rapidly and almost inevitably lead to declines in vaccination coverage.

In both Israel and the occupied Palestinian Territory, past responses to polio involved a collaborative approach. Following the 1988 polio outbreak, a joint early warning system was set up to track sewage from Israel, the West Bank, and Gaza, managed by Israel's Central Virus Laboratory. The WHO recognized Israel as polio-free in 2002, followed by the West Bank and Gaza in 2010 (10, 22).

Two factors related to the war in Gaza, both risk factors for polio, have contributed to the detection of polio cases and the presence of the virus in sewage from samples collected in June and confirmed in July 2024. Firstly, the conflict-related disruption of routine immunization services resulted in a sharp drop in polio vaccination coverage in Gaza, from 99.6% in 2022 (before 7 October 2023) (10) to <90% in the first quarter of 2024 (11, 23), which is below the herd immunity threshold necessary to halt polio transmission. Secondly, before the conflict, an estimated 98% of waste was effectively managed through Gaza's disposal system; this rate has since plummeted to just 20%, leading to overflowing landfill sites and uncollected garbage. Additionally, the safe water supply has diminished by approximately 95% (10, 12, 24). In the geographically close Middle East region, immunization and WASH deficiencies create conditions conducive to spreading disease across national borders. So it is not surprising that the polio virus detected in Gaza was genetically linked to a virus detected in Egypt in 2023 (25).

Despite Israel's polio vaccination coverage of 99%, there remain pockets of unvaccinated children due to religious exemptions, particularly the Ultra-Orthodox Jewish community. After polio virus was found in sewage in Gaza, news channels reported a Health Ministry statistic indicating that at least 175,000 Israeli children are unvaccinated against polio, raising concerns of a potential polio outbreak (11). The Israel Defense Forces responded by offering polio vaccination to soldiers returning from Gaza (11).

These concerns may also be linked to the two recent polio outbreaks in Israel in 2022 and 2023. Vaccine-derived poliovirus type 3 (cVDPV3) was found in routine sewage samples in 2021 and early 2022, and a case of acute flaccid paralysis was reported in a child, later confirmed as caused by cVDPV3 (26). Six more asymptomatic VDPV3-positive cases were confirmed in April 2022. Genetic testing linked this strain to VDPV3 found in samples from Israel and the occupied Palestinian Territory between September 2021 and January 2022 (27). While there was no evidence of circulation in the Palestinian Territory, frequent cross-border movements heighten the risk for unvaccinated children on both sides. In April 2022, another strain (cVDPV2) was detected, initially in Jerusalem, with a clinical case reported in February 2023 (26).

The high risk of transmission is perpetuated by the ongoing conflict. Following the interruption of polio vaccination in Gaza in 2024, a 17-year-old from Jerusalem, who lacked a vaccination history, was diagnosed with polio in December 2024 (https://www.gov.il/en/pages/25122024-02, accessed 28 May 2025). Routine sewage inspections continued to indicate the presence of poliovirus in the area, although no new cases were reported as of May 2025 (https://www.gov.il/en/pages/24042025-02, accessed 28 May 2025).

### International institutional support, including the role of GPEI

With a dedication to eradicating polio worldwide, the GPEI was established in 1988 as a public-private partnership led by six partners: the World Health Organization (WHO), Rotary International, the US Centers for Disease Control and Prevention (CDC), UNICEF, the Gates Foundation, and Gavi, the Vaccine Alliance. The involvement of major international players and the focus on a single goal, backed by billions of US-dollars in investments, expedites high-level efforts for advocacy and diplomacy even in fragile, conflict-affected countries. Able to claim neutrality, GPEI engages with various warring parties, including NSAGs and parties governments refuse to negotiate with. UN agencies led most of these vaccination pauses. For example, in 1999, Kofi Annan, the UN Secretary-General, led negotiations for “days of tranquility” in the Democratic Republic of the Congo to support a polio immunization campaign targeting around 10 million children (28). GPEI also led advocacy initiatives to collect political support for polio vaccination in politically fragile settings. For example, during the polio vaccination boycott in northern Nigeria in 2003, the GPEI secretariat at WHO managed to engage the Organization of the Islamic Conference (OIC) as a mediator to help restore vaccinations in the Muslim-dominant region (29).

**Figure 1:**
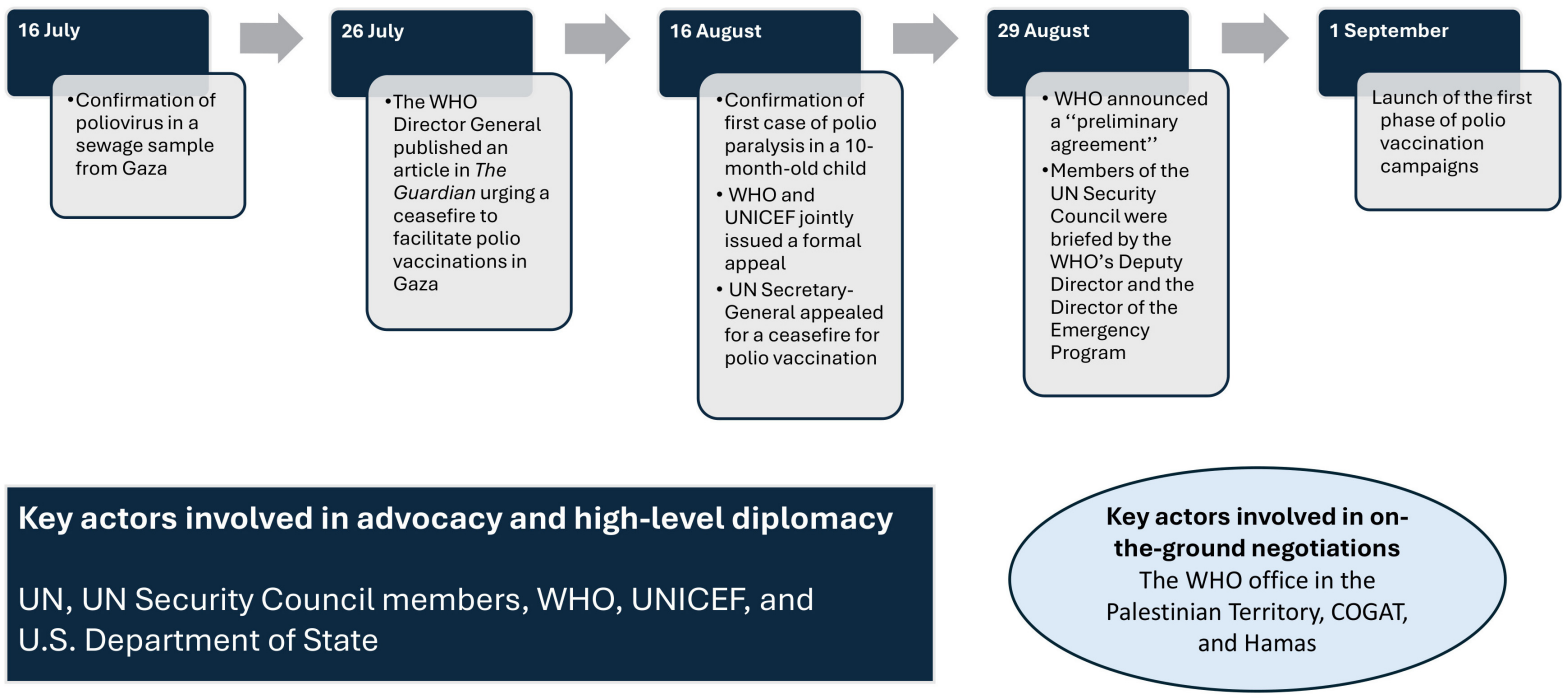
Timeline showing key actors and critical events leading to the first polio vaccination campaign in Gaza, 2024.

The negotiation process for the 2024 polio vaccination pause in Gaza, though, was not publicly documented; we attempted to track the main relevant activities of global health diplomacy during conflict, with the key role of the GPEI, particularly its lead actors: the WHO and UNICEF. This effort unfolded through a coordinated advocacy campaign combining diplomacy, public appeals, and strategic negotiation, culminating in a humanitarian pause that allowed the first polio vaccination campaign in Gaza in September 2024 (see Figure 1).

On 16 July 2024, cVDPV was confirmed in the sewage of Gaza, after collecting six sewage samples in June 2024 from two areas: Khan Younis and Deir al-Balah (30). Ten days later, on 26 July 2024, the WHO Director-General, Dr. Tedros, published an article in The Guardian urging a ceasefire to facilitate emergency vaccinations in Gaza (31). This public call was among various advocacy efforts by WHO and UNICEF, which also included statements to the UN Security Council, media briefings, and diplomatic outreach to member states (32). On August 16, 2024, a heightened urgency arose as Gaza's Ministry of Health confirmed its first clinical case of polio paralysis in a 10-month-old child—the first in Gaza in over 25 years. In response to this alarming development, the WHO and UNICEF jointly issued a formal appeal for a seven-day humanitarian pause from “all parties to the conflict” (25). This pause would facilitate two rounds of polio vaccination aimed at over 640,000 children below the age of ten years. UN Secretary-General António Guterres reinforced this appeal, emphasizing that a vaccination campaign would be unattainable without a cessation of hostilities (33).

The direct negotiation process involved coordination at various levels, with high-level diplomatic pressure. For example, U.S. Secretary of State Antony Blinken reportedly raised the issue directly with Israeli officials during a visit. He demanded that Israel permit vaccination pauses (34), which likely constituted a decisive factor in Israel's commitment. In parallel, the WHO's country office for the occupied Palestinian Territory engaged in on-the-ground negotiations with the Israeli Coordinator of Government Activities in the Territories (COGAT) and secured a preliminary agreement (35). Humanitarian intermediaries and UN channels pursued discussions with Hamas authorities. On 29 August 2024, the WHO announced a “preliminary agreement” with both Israel and Hamas to implement daily nine-hour pauses in specific zones to allow polio vaccinations to proceed. The first round of vaccinations was scheduled to begin in early September 2024. Meanwhile, WHO continued to categorize polio as a Public Health Emergency of International Concern (PHEIC) (26), and WHO Europe (to which Israel belongs) stresses the need for constant surveillance and increased vaccine uptake in the region (16).

### Brief required pause in hostilities, vertical nature, and straightforward impact assessment

Polio vaccination campaigns in conflict settings are carried out in defined areas during specific “days of tranquility” (36). These are essentially brief pauses in hostilities aimed at facilitating vertically organized vaccination efforts within the brief time periods required, rather than long pauses or a complete ceasefire. The conflicting parties understand that once the vaccination days conclude, hostilities will resume, meaning they are not permanently yielding territory or any strategic advantage. Campaign outcomes are relatively easily promptly measurable (number of children vaccinated, cases prevented), allowing for a clear demonstration of impact and accountability to stakeholders and funders.

In Gaza, the polio campaigns were designed around specific locations and times as Israel agreed for daily eight-hour pauses in hostilities (from 6 am to 3 pm) over three-day intervals. The agreement allowed three zoned pauses in fighting for the first round of vaccinations, starting in central Gaza, then moving to southern Gaza, and finally to northern Gaza (34). UN and healthcare officials, including Rik Peeperkorn, WHO representative in the Palestinian Territory, remarked that this is not the ideal solution, but it does present a feasible path forward. Leaders of humanitarian organizations, including the head of the United Nations Relief and Works Agency for Palestine Refugees in the Near East (UNRWA), emphasized that these “polio pauses” provided temporary relief during the crisis while advocating for an extended ceasefire (23, 35).

### Perceived “neutrality”: children as the primary focus of polio vaccination

Vaccination programs are primarily targeted at children, which also applies to polio vaccination campaigns during conflicts (36). Thus, polio campaigns are generally perceived as neutral and humanitarian, making it politically easier for warring parties to agree to temporary pauses without appearing to cede strategic ground: Adversaries share an understanding regarding the “need to secure their children's future” (37), acknowledging that these innocent children bear no responsibility for the war. This has been observed even after attacks eliciting strong negative emotional responses toward an adversary, as is the case after the October 7 attacks (38). The argument of children as beneficiaries could as well be made for improving WASH, as UNICEF does (2). Improving WASH, however, like supplying food, can be construed as also benefitting (adult) fighters, a position repeatedly taken by representatives of Israel's Netanyahu government, although withholding water or food from civilians violates international law (39). As improving WASH involves control over critical resources such as water pipelines, which can be leveraged as tools of war or negotiation, parties may be less willing to relinquish control or allow repairs that could strengthen the adversary.

## Discussion: comparing vaccination campaigns and WASH

Water and sanitation are pressing daily needs felt by each person and family. As opposed to vaccination campaigns which can be vertically organized, WASH needs more permanent infrastructure and collaboration across different sectors to work. In low-income settings, provision may be decentralized, for example based on local wells and latrines. In areas without direct military action, they may remain intact. Armed conflicts in settings with highly centralized services lead to different scenarios. Gaza, for example, received a substantial part of its drinking water from Israel via three main pipes (12). Control over providing, or withholding, safe drinking water and sanitation measures thus becomes a simple and easily controllable tool to put pressure on an adversary, and to withhold vital resources from fighters. In March 2025, Israel has openly threatened to again reduce, or even close, water supply to the civilian population of Gaza unless Hamas releases the remaining hostages (40), in clear violation of international humanitarian law. This could explain the limited role of WASH in mitigating acute conflict and instigating humanitarian pauses. Moreover, the preference of short-term vaccination campaigns over longer-term investment in WASH reflects a general tendency to favor vertical over horizontal interventions with easily measurable outcomes in disease control (41). WASH improvements are harder to attribute directly to health outcomes in the short term and require ongoing monitoring, making it more challenging to justify and sustain interventions in unstable settings. We have summarized these arguments in Table 1.

**Table 1:** Comparison of vaccination campaigns and WASH improvements.

**Parameter compared**	**Polio vaccination campaigns**	WASH^*^improvements
Dimension	Vertical	Horizontal
Scope and complexity	Targeted, short-term, low infrastructural needs	Broad, long-term, high infrastructural needs
Political acceptability	Neutral, focused on children	Involves strategic resources
International Support	Strong, unified, high-level	Diffuse, less coordinated
Measurability	Immediate, specific, and thus countable outcomes	Long-term, diverse benefits
Security and logistics	Brief, mobile, less exposed	Prolonged, stationary, high risk
Symbolic value	High (children's health)	Lower, less emotionally mobilizing
Duration of commitment	Short, defined	Long, open-ended

^*^WASH, Water, sanitation and hygiene. Here in particular: provision of safe drinking water, collection and treatment of sewage.

WASH may play a more important and successful role in long-term peace making and re-establishing collaboration between opponents (1). Immunization may appear, in comparison to WASH, as a less pressing need at the individual level; but low coverage has public health implications such as disease outbreaks that may reach further (i.e., affect larger geographical areas) and are therefore more difficult to control. Moreover, unlike the control of water supply, conflict parties cannot instrumentalize epidemic outbreaks in a targeted or dosed way. A similar argument applies to vaccination: for antigens such as measles or polio, reaching the herd immunity level is paramount from the point of view of all sides involved.

## Future perspectives

The specifics surrounding how parties to a conflict agreed to enter negotiations about vaccination pauses remain inadequately documented and require further research. Of interest is also why measles, a highly contagious disease with a substantial case fatality rate in malnourished children in resource-poor settings (42), receives less attention than polio in spite of WHO having raised concerns (23). The future of polio vaccination worldwide, particularly in war-torn countries, remains uncertain due to the escalating armed conflicts and the decisions of the U.S. administration in 2025 to withdraw from WHO, dismantle USAID, and reduce support for the CDC, which are major actors in the polio eradication efforts and humanitarian aid (43).

On a positive note, fostering a mutual understanding of public health perspectives is vital for facilitating effective dialogue in conflict situations, particularly concerning vaccination negotiations. While these discussions may initially have a limited impact on trust, their long-term effects can be profound, especially in the aftermath of conflict (36). Acknowledging the life-saving benefits of vaccinations can help both parties recognize their shared humanity and the value of collaborative efforts in rebuilding trust and promoting health in the future. Thus, while consensus may not be guaranteed, the potential for improved relations and outcomes through a public health lens remains significant. Public health efforts should seize every opportunity to foster cooperation between warring parties. Such initiatives—be they in vaccination, WASH, or working together academically—can help build trust, laying the groundwork for future peace negotiations and post-conflict reconstruction (10, 12).

## Data Availability

The original contributions presented in the study are included in the article/supplementary material, further inquiries can be directed to the corresponding author.
